# Axonal α7* nicotinic acetylcholine receptors modulate glutamatergic signaling and synaptic vesicle organization in ventral hippocampal projections

**DOI:** 10.3389/fncir.2022.978837

**Published:** 2022-09-23

**Authors:** Chongbo Zhong, Wendy Akmentin, Lorna W. Role, David A. Talmage

**Affiliations:** ^1^National Institute of Neurological Disorders and Stroke, National Institutes of Health (NIH), Bethesda, MD, United States; ^2^Department of Neurobiology and Behavior, Center for Nervous System Disorders, Stony Brook University, Stony Brook, NY, United States

**Keywords:** α7* nicotinic acetylcholine receptor, glutamatergic transmission, cholinergic modulation, neurotransmitter release, synaptic vesicle fusion, optogenetics, electron microscopy, iGluSnFr

## Abstract

Modulation of the release of glutamate by activation of presynaptic nicotinic acetylcholine receptors (nAChRs) is one of the most prevalent mechanism of nicotinic facilitation of glutamatergic transmission in cortico-limbic circuits. By imaging gene chimeric co-cultures from mouse, we examined the role of α7* nAChRs mediated cholinergic modulation of glutamate release and synaptic vesicle organization in ventral hippocampal projections. We directly visualized exogenous and endogenous cholinergic facilitation of glutamate release in this specialized preparation of circuits *in vitro*. Disrupting α7* nAChRs mediated cholinergic signaling genetically or pharmacologically diminished cholinergic facilitation of glutamate release at presynaptic terminals. Alteration of α7* nAChRs mediated cholinergic signaling along glutamatergic axons also decreased functional synaptic vesicle clustering to presynaptic terminals. These findings suggest that presynaptic α7* nAChRs contribute to cholinergic modulation of glutamate release and synaptic vesicle organization.

## Introduction

Cholinergic signaling plays critical functions in the mammalian brain, contributing to attention, wakefulness and the formation and recall of memories (Ballinger et al., 2016). The importance of these functions is underscored by the connections between disruption in the cholinergic system and conditions ranging from thought disorders to cognitive impairment and dementia. Understanding how cholinergic inputs influence various forebrain circuits is essential to defining the role of acetylcholine in these conditions (Freedman et al., 2001; Mathew et al., 2007; Barreto et al., 2015; Lim et al., 2015; Alber et al., 2020; Bohnen et al., 2022; Polli and Kohlmeier, 2022).

Cholinergic projection neurons of the basal forebrain and brainstem send axons to virtually all regions of the brain where they branch extensively allowing a relatively small number of neurons to contribute to an array of critical functions. Acetylcholine acts *via* two general classes of receptors, the G-protein coupled muscarinic receptors (mAChRs) and the ligand gated cation channel nicotinic receptors (nAChRs). The most abundant nAChRs in the brain are the α4β2* and α7* nAChRs (the nicotinic ACh receptors are pentamers comprised of all alpha or combinations of alpha and beta subunits. We use the convention of αβ* and α* to indicate that the subunit stoichiometry is not known) (Kutlu and Gould, 2015; Wittenberg et al., 2020). α4β2* and α7* nAChRs are found at pre-synaptic as well as post-/extra-synaptic sites (Jones and Wonnacott, 2004; Zhong et al., 2008; Cheng and Yakel, 2014). Activation of axonal and/or pre-synaptic nAChRs affects the release of many neurotransmitters including glutamate, dopamine, GABA and even ACh (McGehee et al., 1995; Gray et al., 1996; Kramer et al., 2022; Liu et al., 2022). nAChR activity also is linked to synapse development and maturation (Berg and Conroy, 2002; Lozada et al., 2012). Taken together, there is strong evidence that nAChRs play multiple roles in the formation and function of brain circuits.

Axonal α7*nAChRs can increase glutamate release probabilities by elevating intracellular Ca^2+^-signaling *via* multiple mechanisms. First, α7*nAChR activation leads to local depolarization and opening of voltage dependent calcium channels. Second, α7* nAChRs are calcium permeable, allowing rapid and local increases in intracellular calcium. When localized close enough to neurotransmitter release sites, α7*nAChR gated calcium transients contribute to synaptic vesicle exocytosis, increasing the probability of transmitter release (Fucile, 2004; Turner, 2004; Fayuk and Yakel, 2005; Shen and Yakel, 2009; Cheng and Yakel, 2015a,b; Cheng et al., 2021).

Activation of axonal α7*nAChRs also leads to sustained increases in intracellular calcium signaling; a response linked to the coupling of ligand-activated α7*nAChRs to heterotrimeric G proteins (Zhong et al., 2013; Nordman and Kabbani, 2014; King et al., 2015). The ability of α7*nAChRs to activate second messenger signaling allows these receptors to regulate axonal actions ranging from growth cone dynamics to sustained glutamate release (Nordman and Kabbani, 2012; Kabbani and Nichols, 2018). Prior studies have predominantly used pharmacological agents to probe axonal α7*nAChR function leaving open the question to the extent to which axo-axonal interactions between cholinergic axons and target axons elicit similar α7*nAChR dependent signaling.

Glutamatergic synaptic transmission requires calcium dependent release of glutamate from presynaptic terminals. Although glutamatergic transmission, and its modulation by pre-synaptic nAChRs, has been studied in detail at individual synapses electrophysiologically, optical reporters of synaptic vesicle recycling and neurotransmitter release allow direct measurement of presynaptic phenomena, allowing us to address questions related to the effects of α7* nAChR signaling on the dynamics of synaptic vesicular cycling (Liu and Tsien, 1995; Moulder et al., 2006; Alabi and Tsien, 2012; Marvin et al., 2013, 2018; Zhong et al., 2015, 2017; Helassa et al., 2018).

In this study we used a variety of imaging modalities to examine exogenous and endogenous cholinergic modulation of glutamate release and synaptic vesicle recycling in ventral hippocampal glutamatergic projections using gene-chimeric micro-slice co-cultures. We provide evidence for two distinct functions of axonal α7* nAChR signaling. The first involves sustained increases in glutamate release in response to both levels of nicotine approximating those delivered to the CNS by smoking and by levels of acetylcholine released from basal forebrain cholinergic neurons. The second function for axonal α7* nAChRs is in the formation of functional glutamate release sites by contributing to the organization of synaptic vesicles.

## Materials and methods

### Materials

#### Mice

C57Bl6/J (000668), B6.129S7-Chrna7^*TM*1*Bay*^/J (003232) and B6;129S6-Chat^*TM*2(*cre*)*Lowl*^/J (006410) mice were obtained from Jackson Labs, the Chat-tau-eGFP BAC transgenic line was a gift from Dr. S. Vijayarhagavan, U. Colorado (Grybko et al., 2011). All mice were maintained on 12 h light: 12 h dark cycles with constant temperature (72°F) and humidity (40%). Mice were allowed *ad libitum* access to food and water. All animal handling was done in accordance with the National Institutes of Health Guide for the Care and Use of Laboratory Animals (NIH Publications No. 80-23, revised 2012) and was approved by either the Stony Brook University Institutional Animal Care and Use Committee (#1618) or the NINDS ACUC (ASP#1490).

#### Viruses

Packaged preparations of AAV_9_.hSyn.iGluSnFr.WPRE. SV40 (gift from Loren Looger) and AAV_9_-EF1a-DIO-C1V1(E122T/E162T)-TS-mCherry (gift from Karl Deisseroth) were obtained from Addgene (Addgene viral prep #98929-AAV9; Addgene_98929) or the University of North Carolina viral vector core, respectively.

#### Antibodies and other reagents

vGluT1 (Synaptic systems, #135 303), pan-axonal neurofilament marker (SMI312, Sigma-Aldrich, # NE1022), Synapsin 1 (EMD Millipore, #1543), phospho (Ser62, Ser67) Synapsin 1 (EMD Millipore, #AB9848), alpha-bungarotoxin (Sigma-Aldrich, #B137), alpha-bungarotoxin-594 (Thermo Fisher #B-13423), FM1-43 (Thermo Fisher #T35356), Fluo4 (Thermo Fisher #F14217), tetrodotoxin (Tocris #1078), bicuculline (Tocris #0130), RuBi-nicotine (Tocris, #3855), D-AP-5 (Tocris #0106), and CNQX (Tocris #1045).

### Methods

#### Micro-slice cultures

The cultures were prepared as described previously (Zhong et al., 2015). Briefly, for ventral hippocampal micro-slice cultures, the ventral CA1 and subiculum area (vHipp) from WT (+/+) or α7 knockout (−/−) mice (postnatal days 0–3, P0–P3) was dissected, sliced into ∼150 μm × 150 μm pieces, and then plated onto poly-D-lysine/laminin-coated glass coverslips (12 mm, BD Sciences) in 24-well plates with a minimal volume (50 μl) of culture media [Neurobasal, 2% B-27 (GIBCO) and 20 ng/ml of brain-derived neurotrophic factor (Invitrogen)]. After the micro-slices attached to the substrate, 100 μl of additional culture media was added. For medial septum/diagonal band (MS/DB) cultures, the MS/DB area (Bakker et al., 2015; Staib et al., 2018) from ChAT-tau-eGFP or ChAT-IRS-Cre mice (P0–P3) was dissected, sliced and plated on coverslips as described before (Zhong et al., 2015) and above for vHipp cultures.

For vHipp-nucleus accumbens (nAcc) synaptic co-cultures, nAcc (ED18-P1) from WT mice (C57BL/6J) were dissected out and dispersed with 0.25% trypsin (GIBCO) for 15 min at 37°C, followed by gentle trituration in culture media. Dispersed nAcc neurons were plated onto poly-D-lysine/laminin-coated glass coverslips at 0.25 ml/coverslip. The next day vHipp micro-explants [prepared as described above and in Zhong et al. (2008)] were added to the same coverslips.

### AAV infections

The genetically encoded glutamate indicator, iGluSnFr or the red-shifted opsin, C1V1, were expressed by adding AAV-hSyn-iGluSnFr-WPRE-SV40 (10^13^ vg/ml) or AAV-Ef1a-DIO C1V1 (t/t)-TS-mCherry (2 × 10^12^ vg/ml) directly to the coverslip (0.25 μl/well). Twenty-four hours later the culture media was changed and replaced with virus-free media. All cultures were maintained in a humidified 37°C, 5% CO_2_ incubator 7–10 days post-infection before performing imaging experiments. For co-culture experiments involving vHipp micro-slices and dispersed nAcc neurons, the nAcc neurons were plated and infected first and vHipp micro-slices were added to the same coverslips 24 h later following virus wash-out. For vHipp – MS/DB co-cultures, vHipp explants from C57Bl6/J mice were plated and infected with AAV-hSyn-iGluSnFr-WPRE-SV40 on day 1 and MS/DB micro-slices from Chat-IRES-Cre mice were plated on the same coverslips 24 h later. Following plating of the MS/DB micro-slices, AAV-Ef1a-DIO C1V1 (t/t)-TS-mCherry was added to the coverslips and infection allowed to proceed for 24 h following which the culture media was replaced. In this way, iGluSnFr and C1V1 expression were limited to vHipp projections or MS cholinergic neurons, respectively.

### FM1-43-based live imaging and analysis

After 7–10 days *in vitro*, coverslips with cultures were transferred and sealed to the bottom of an imaging Ludin chamber (Live Imaging Services, Switzerland) and then mounted on an Olympus IX81 DSU microscope (spinning disk confocal, Olympus America) under continuous superfusion (1 ml/min) with HEPES buffered saline (HBS, 135 mM NaCl, 5 mM KCl, 1 mM MgCl_2_, 2 mM CaCl_2_, 10 mM HEPES, and 10 mM glucose, pH 7.35) containing 2 μM tetrodotoxin (TTX), 10 μM bicuculline, 50 μM D-AP-5, and 20 μM CNQX. KCl induced depolarization (Amaral et al., 2011) was used for loading FM1-43 into axons. Cultures were loaded with 10 μM FM1-43 in 56 mM KCl ACSF for 90 s, external dye was washed away in Ca^2+^-free HBS, containing ADVASEP-7 (0.1 mM; Sigma, # A3723, to scavenge membrane-bound FM1-43) for 15 min. Fluorescence images of vHipp and/or MS/DB axons were collected by a Plan-Apochromat objective (60× oil with 1.4 NA, excitation 488 nm, emission 530 nm) and captured with a CCD camera (Hamamatsu) every 1.5 s for 5 min. Image acquisition was performed using Slidebook software (version 5; Olympus). After 1 min of baseline data collection, 56 mM KCl in HBS without FM1-43 was applied for 120 s to confirm that high K^+^ depolarization induced de-staining of FM1-43 dye–filled vesicles. The total amount of releasable FM1-43 fluorescence at each synaptic bouton was calculated from the difference between fluorescence intensity after staining (but before de-staining) and after nicotine (1 μM) application (applied by rapid perfusion (2 ml/min) for 1 min), KCl depolarization, or opto-stimulation (100 total 5 ms pulses at 10 Hz) induced de-staining (Δ*F* = *F*_staining_ − *F*_de–staining_). The fraction of fluorescence intensity decrease after depolarization was calculated as *F*_decrease_% = Δ*F*/*F*_staining_. The number and size of FM1-43 positive puncta before and after nicotine application, or KCl depolarization was measured and compared along vHipp axons from α7+/+ wild type vs. α7−/− knockout mice using MetaMorph software. The lengths of axonal projections were also measured by tracing vHipp and/or MS projections in MetaMorph.

For analyzing the time course of nicotine induced FM1-43 de-staining, all frames of the raw FM1-43 fluorescence images were saved as Slidebook files and then exported as a series of TIF format images that were then imported to MetaMorph software and transferred as Z-stack images for further analyses. After setting the threshold of the FM1-43 fluorescence, the integrated intensity of the FM1-43 signals along vHipp axons before and after nicotine application was calculated. FM1-43 fluorescence data are displayed as a normalized integrated intensity: Δ*F*/*F*_0_ = (*F* − *F*_0_)/*F*_0_, where *F*_0_ is the background-corrected pre-nicotine FM1-43 fluorescence. Data were analyzed further using Excel and GraphPad Prism 9 software.

### iGluSnFr-based live imaging and analysis

After 7–10 days *in vitro*, cultures expressing iGluSnFr were transferred to an imaging chamber and continuously superfused with HBS cocktail as described above for FM1-43 live imaging. Fluorescence images of iGluSnFr under various culture conditions were collected every 2 s for 20 min. After 1–2 min of baseline data collection, glutamate (100 μM), or nicotine (1 μM) was applied by rapid perfusion (2 ml/min) for 1 min. For testing the effect of endogenous ACh release on glutamate release, C1V1 expressing MS/DB projections were stimulated with a 594 nm LED light (5 ms pulse duration, 10 Hz for 10 s) after 2 min baseline data collection.

All frames of the raw iGluSnFr fluorescence images were saved as Slidebook files and then exported as a series of TIF format images that were then imported to MetaMorph software and transferred as Z-stack images for further analyses. After setting the threshold of the iGluSnFr fluorescence, the integrated intensity of the fluorescence signals before and after glutamate or nicotine application or following opto-stimulation were collected and calculated. Fluorescence data are displayed as a normalized integrated intensity: [Δ*F*/*F_0_* = (*F* −*F_0_*)*/F_0_*], where *F*_0_ is the background-corrected pre-stimulation iGluSnFr fluorescence. Data were analyzed further using Excel software. The iGluSnFr data from vHipp-nAcc and MS/DB-vHipp synaptic co-cultures are displayed in box plots (Statview and GraphPad Prism 9 software) where the boxes include data points between the twenty-fifth percentile (bottom line) and the seventy-fifth percentile (top line). The middle line indicates the fiftieth percentile (median). Vertical lines mark the fifth and ninety-fifth percentiles.

### Electron microscopy

After 7–10 days *in vitro*, vHipp micro-slices grown on Thermanox plastic coverslips (NUNC/Electron Microscopy Sciences) were fixed for 30 min in a mixture of cold 2% paraformaldehyde and 2% glutaraldehyde in 0.1 M phosphate buffer (PB), pH 7.4. After several washes in PB, explants were postfixed with 2% osmium tetroxide for 30 min, en bloc stained with aqueous 1% uranyl acetate for 30 min, dehydrated through an ascending series of ethanol concentrations, followed by acetonitrile, and embedded in Embed 812 resin (Electron Microscopy Sciences) for 48 h at 60°C. Ultrathin sections (60–90 nm) were cut and then stained with 1% methanolic uranyl acetate and 0.3% aqueous lead citrate, and observed with a JEOL 1200EX transmission electron microscope.

### Immunocytochemistry and reagents

After 7–10 days *in vitro*, cultures were fixed in 4% paraformaldehyde/4% sucrose/PBS [20 min, room temperature (RT)], permeabilized with 0.25% Triton X-100/PBS (5 min, RT), blocked with 10% normal donkey serum in PBS (30 min, RT), and then incubated in primary antibodies overnight at 4°C. The following primary antibodies were used: anti-vesicular glutamate transporter 1 (1:250), anti-pan-axonal neurofilament marker (1:1000), anti-synapsin1 (1:500), and anti-phospho-Synapsin1 (1:500). Cultures were washed and incubated in secondary antibodies conjugated to Alexa Fluor 488 (1:500) or Alexa Fluor 594 (1:500) for 1 h at RT. Slips were mounted using VectaShield (with DAPI, Vector Laboratories), and images were captured using a microscope (Axio Imager A1; Carl Zeiss) equipped with Plan-Apochromat objectives (63× oil with 1.4 NA), a CCD camera (Hamamatsu), and MetaMorph software (version 7.1, MetaMorph Microscopy Automation and Image Analysis Software, Molecular Devices). To label surface α7* nAChRs after live imaging of nicotine-induced FM1-43 de-staining, vHipp cultures were incubated in αBgTx conjugated to Alexa 594 (1:1000; Molecular Probe) for 45 min at RT prior to fixation.

### Statistical analysis

All data were tested for normality with the D’Agostino-Pearson test and expressed as mean ± SEM unless otherwise indicated and analyzed with StatView (SAS Institute), Microsoft Excel (Microsoft) and Prism 9 (GraphPad by Dotmatics) software. Data sampling units and *p*-values correspond to the statistical tests for all data collected are shown in Table 1.

**TABLE 1:** Statistical analyses used in this study.

Figures	Data sampling units	Type of test	*P*
Figure 1C traces with Mean and shaded Standard Deviation	40 axons from 5 separate experiments for α7+/+ 40 axons from 4 separate experiments for α7−/−		
Figure 1D, FM1-43 decrease %, α7+/+ vs. α7−/−	100 axons from 5 separate experiments for α7+/+ 92 axons from 4 separate experiments for α7−/−	Unpaired Student’s *t*-test; *t* = 28.04	<0.001*
Figure 1E, FM1-43 decay time, α7+/+ vs. α7−/−	52 axons from 5 separate experiments for α7+/+ 46 axons from 4 separate experiments for α7−/−	Unpaired Student’s *t*-test; *t* = 41.35	<0.001*
Figure 2D traces with Mean and shaded Standard Deviation	30 αBgTX^+^ and 20 αBgTX^–^ puncta from 3 separate experiments		
Figure 2E, FM1-43 decrease %, αBgTX^+^ vs. αBgTX^–^ and α7−/−	60 αBgTX^+^ and 60 αBgTX^–^ puncta from 3 separate experiments with *post hoc* surface αBgTX-Alexa 594 labeling; 46 axons from 4 separate experiments for α7−/−	One-way ANOVA; *F*(2,163) = 413.5	<0.001*
Figure 2E, FM1-43 decrease %, αBgTX^–^ vs. α7−/−	60 αBgTX^–^ puncta from 3 separate experiments with *post hoc* surface αBgTX-Alexa 594 labeling; 46 axons from 4 separate experiments for α7−/−	One-way ANOVA, Tukey’s *post hoc* test	0.179*
Figure 2F, FM1-43 decay time, αBgTX^+^ vs. αBgTX^–^ and α7−/−	60 αBgTX^+^ and 60 αBgTX^–^ puncta from 3 separate experiments with *post hoc* surface αBgTX-Alexa 594 labeling; 46 axons from 4 separate experiments for α7−/−	One-way ANOVA; *F*(2,163) = 468.8	<0.001*
Figure 2F, FM1-43 decay time, αBgTX^–^ vs. α7−/−	60 αBgTX^–^ puncta from 3 separate experiments with *post hoc* surface αBgTX-Alexa 594 labeling; 46 axons from 4 separate experiments for α7−/−	One-way ANOVA Tukey’s *post hoc* test	0.2321*
Figure 3C, Ratio of intensities, WT vs. 7 days αBgTX	20 axons (>1,500 μm in total axon length) from 3 separate experiments for each group	One-way ANOVA *F*(3,76) = 70.91, Tukey’s *post hoc*	0.9512*
Figure 3C, Ratio of intensities, WT vs. WT + Nic	20 axons (>1,500 μm in total axon length) from 3 separate experiments for each group	One-way ANOVA Tukey’s *post hoc* test	<0.001*
Figure 3C, Ratio of intensities, 7 days αBgTX vs. 7 days αBgTX + Nic	20 axons (>1,500 μm in total axon length) from 3 separate experiments for each group	One-way ANOVA Tukey’s *post hoc* test	0.9686*
Figure 3D, Ratio of intensities, WT vs. 7 days αBgTX	20 axons (>1,500 μm in total axon length) from 3 separate experiments for each group	One-way ANOVA *F*(3.76) = 120.8 Tukey’s *post hoc* test	0.9998*
Figure 3D, Ratio of intensities, WT vs. WT + Nic	20 axons (>1,500 μm in total axon length) from 3 separate experiments for each group	One-way ANOVA Tukey’s *post hoc* test	0.2265*
Figure 3D, Ratio of intensities, 7 days αBgTX vs. 7 days αBgTX + Nic	20 axons (>1,500 μm in total axon length) from 3 separate experiments for each group	One-way ANOVA Tukey’s *post hoc* test	0.9215*
Figure 4C traces with Mean and shaded Standard Deviation	6 neurons/condition from 3 separate experiments with +/+ to +/+ co-cultures 5 neurons/condition from 4 separate experiments with −/− to +/+ co-cultures		
Figure 4D, Control iGluSnFr +/+ to +/+ vs. −/− to +/+	8–11 neurons/condition from 4 separate experiments	One-way ANOVA *F*(3,34) = 20.77; Tukey’s *post hoc* test	0.9513*
Figure 4D, +/+ to +/+ iGluSnFr control vs. +Nic	8 neurons/condition from 3 separate experiments with +/+ to +/+ co-cultures	One-way ANOVA Tukey’s *post hoc* test	<0.001*
Figure 4D, −**/**− to +/+ iGluSnFr control vs. +Nic	11 neurons/condition from 4 separate experiments with −/− to +/+ co-cultures	One-way ANOVA Tukey’s *post hoc* test	0.9469*
Figure 5B traces with Mean and shaded Standard Deviation	5 axons from 2 separate experiments		
Figure 5C traces with Mean and shaded Standard Deviation	20 axons from 3 separate experiments for each group		
Figure 5D, Control iGluSnFr WT vs. WT + αBgTX	20 axons (>1,500 μm in total axon length) from 3 separate experiments for each group	One-way ANOVA *F*(5,114) = 197.0, Tukey’s *post hoc* test	0.6512^#^>
Figure 5D, WT iGluSnFr control vs. 1′ post Nic and 20′ post Nic	20 axons (>1,500 μm in total axon length) from 3 separate experiments for each group	One-way ANOVA Tukey’s *post hoc* test	<0.001 (1′)^#^>*CPSTABLEENTER* < 0.001 (20′)^#^>
Figure 5D, WT + αBgTX iGluSnFr control vs. 1′ post Nic and 20′ post Nic	20 axons (>1,500 μm in total axon length) from 3 separate experiments for each group	One-way ANOVA Tukey’s *post hoc* test	0.7024(1′) ^#^> 0.8763 (20′)^#^>
Figure 6D, Fluo-4, pre vs. post light	20 axons (>1,000 μm in total axon length) from 4 separate experiments	Unpaired Student’s *t*-test, *t* = 16.81	<0.001*
Figure 6D, FM1-43, pre vs. post light	20 axons (>1,000 μm in total axon length) from 4 separate experiments	Unpaired Student’s *t*-test, *t* = 23.81	<0.001*
Figure 6D, iGluSnFr, pre vs. post light	20 axons (>1,000 μm in total axon length) from 4 separate experiments	Unpaired Student’s *t*-test, *t* = 0.07679	0.9389^#^>
Figure 7C traces with Mean and shaded Standard Deviation	21 axons from 3 separate experiments for vHipp with close contacts with C1V1 expressed MS/DB axons 9 axons from 3 separate experiments for vHipp with no close contacts with C1V1 expressed MS/DB axons		
Figure 7E, iGluSnFr Control vs. Opto, Atrop + Opto, and MEC + Opto.	30 axons (>1,000 μm in total axon length) from 3 to 6 separate experiments with axon-axon co-cultures for each group	One-way ANOVA, *F*(3,308) = 87.71 Tukey’s *post hoc* test	<0.001 (Opto)* <0.001 (Atrop + Opto)* 0.539 (MEC + Opto)*
Figure 8B, FM1-43 intensity, α7+/+ vs. α7−/−	100 axons from 5 separate experiments for α7+/+ 92 axons from 4 separate experiments for α7−/−	Unpaired Student’s *t*-test, *t* = 0.5589	0.5769*
Figure 8C, FM1-43 cluster numbers, α7+/+ vs. α7−/−	100 axons from 5 separate experiments for α7+/+ 92 axons from 4 separate experiments for α7−/−	Unpaired Student’s *t*-test, *t* = 18.81	<0.001*
Figure 8D, FM1-43 cluster size, α7+/+ vs. α7−/−	100 axons from 5 separate experiments for α7+/+ 92 axons from 4 separate experiments for α7−/−	Unpaired Student’s *t*-test, *t* = 23.62	<0.001^#^>
Figure 8E, FM1-43 decrease % α7+/+ vs. α7−/−	20 axons from 5 separate experiments for α7+/+ 20 axons from 4 separate experiments for α7−/−	Unpaired Student’s *t*-test, *t* = 0.7926	0.4327^#^>
Figure 9B, vesicle cluster numbers, α7+/+ vs. α7−/− and α7+/+ with 7 days αBgTX	20 axons (>200 μm in total axon length) from 2 separate experiments for each group	One-way ANOVA *F*(2,54) = 28.81	<0.001*
Figure 9B, vesicle cluster numbers, α7−/− vs. α7+/+ with 7 days αBgTX	20 axons (>200 μm in total axon length) from 2 separate experiments for each group	One-way ANOVA Tukey’s *post hoc* test	0.3443*
Figure 9C, average vesicle numbers α7+/+ vs. α7−/− and α7+/+ with 7 days αBgTX.	20 axons (>200 μm in total axon length) from 2 separate experiments for each group	One-way ANOVA *F*(2,54) = 12.62	<0.001*
Figure 9C, average vesicle numbers α7−/− vs. α7+/+ with 7 days αBgTX	20 axons (>200 μm in total axon length) from 2 separate experiments for each group	One-way ANOVA Tukey’s *post hoc* test	0.6332*

*Data conformed to normal distribution by the following four tests: Anderson –Darling; D’Agostino and Pearson, Shapiro and Wilk and Kolmogorov–Smirnov test.

^#^Data conformed to normal distribution by the D’Agostino and Pearson and Kolmogorov–Smirnov Test.

## Results

### α7* nicotinic acetylcholine receptors are required for nicotine-induced vesicular release along vHipp axons

**FIGURE 1:**
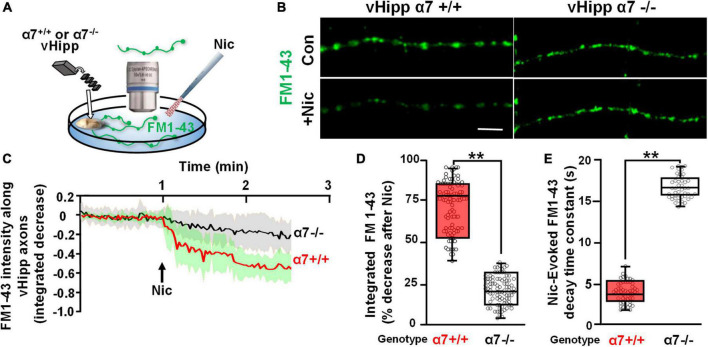
α7* nAChRs are required for nicotine-induced FM1-43 de-staining along ventral hippocampal axons. **(A)** Schematic representation of the experimental configuration. Individual WT (α7+/+) or α7 knock out (α7–/–) mice were used for each plating of ventral hippocampal (vHipp) microexplants. These microexplants, prepared as previously described (Zhong et al., 2015) and subsequently thinned by maintenance in minimal media for an additional 24 h, are represented in the schematic as a tissue-colored oblong volume. The axons that grow out of the microexplant (within the same coverslip) are represented by curving lines (here in green to indicate expression of FM1-43). FM1-43 loading is performed as described in “Materials and methods.” The spatial and temporal dynamics of nicotine-induced release was assayed by high resolution imaging (spinning disk confocal; see details in “Materials and methods”) of FM1-43 de-staining along individual vHipp axons. All drug application was by local micro perfusion as represented in the schematic by a single application pipette. **(B)** Representative micrographs of α7+/+ (**B**, left) and α7–/– (**B**, right) vHipp axons (labeled with FM1-43, green) before (**B**, top) and after (**B**, bottom) nicotine (1 μM, 1 min) application are shown. Scale bar, 10 μm. **(C)** Averaged traces of the FM1-43 fluorescence time course assayed along α7+/+ and α7–/– vHipp axons are shown before and after nicotine application. For the α7+/+ we have plotted the averaged change in fluorescence of 40 independent axon segments of ∼15 μm each, from 5 separate experiments (red line); the standard deviation is shown in green shading. We will use the following notation in the subsequent figure legends: *n* = #axon segment, ## separate experiments. Averaged traces from α7–/– (*n* = 40, 4) before and after nicotine application (black line; SD as gray shaded area). Images collected every 1.5 s for 5 min. FM1-43 fluorescence intensity was calculated and quantified as a normalized integrated intensity at individual 1-μm spots at each time point to yield these representative plots. See Table 1 for sample and statistical details. **(D)** Box plots of the pooled data by condition tested. Box plots are overlaid with all of the data points shown here as a scatter plot. Data for α7+/+ (*n* = 100 axon segments; 5 separate experiments) and α7–/– (*n* = 92 axon segments, 4 separate experiments). The efficacy of nicotine induced neurotransmitter release along vHipp axons was assayed as the percent decrease of overall FM1-43 fluorescence intensity after nicotine application. The extent of nicotine-induced FM1-43 de-staining was significantly lower in vHipp axons from α7–/– mice, compared with the α7+/+ controls (**p < 0.001). All data were first tested for distribution normality by the test(s) listed in Table 1 prior to statistical analysis. See Table 1 for all sample and statistical details. **(E)** Box plots of pooled data by condition tested with overlaid scatter plots of all individual data points) from both α7+/+ and α7–/– vHipp axons. The decay time constant (τ), a dynamic indicator of the rate of nicotine-induced FM1-43 de-staining, was significantly different in α7+/+ vHipp axons (τ∼4 s, 52 axon segments; 5 separate experiments) compared to α7–/– vHipp axons (τ∼16s, 46 axon segments; 4 separate experiments **p < 0.001). All data were first tested for distribution normality by the test(s) listed in Table 1 prior to statistical analysis. See Table 1 for all sample and statistical details of panel **(E)**.

We used FM1-43 de-staining to investigate the dynamics of nicotine induced exocytosis from individual synaptic vesicle clusters along vHipp axons. We used KCl induced depolarization to load vHipp axons from either WT or α7 knockout mice with FM1-43 and then quantified the time course of nicotine-induced FM1-43 exocytosis from individual synaptic vesicle clusters (three pixels, ∼1 μm) by live imaging (every 1.5 s for 5 min) (Figures 1A,B). We plotted the fraction decrease of normalized integrated intensity at single clusters after nicotine application and then calculated the time constants for fluorescence decay along WT (trace in red) and α7 knockout (trace in black) vHipp axons (Figure 1C; traces are averages of recordings along multiple axon segments; the shaded areas around each trace represent standard deviations). Following nicotine application, FM1-43 fluorescence in vesicle clusters along WT vHipp axons decreased by ∼80% with a decay time constant of ∼4 s. In contrast, nicotine only reduced FM1-43 fluorescence by ∼20% with a time constant of ∼16 s from vesicle clusters along α7 knockout axons (Figures 1D,E; WT vs. Knockout, p < 0.001; see Table 1 for additional details regarding n values and statistical analyses).

**FIGURE 2:**
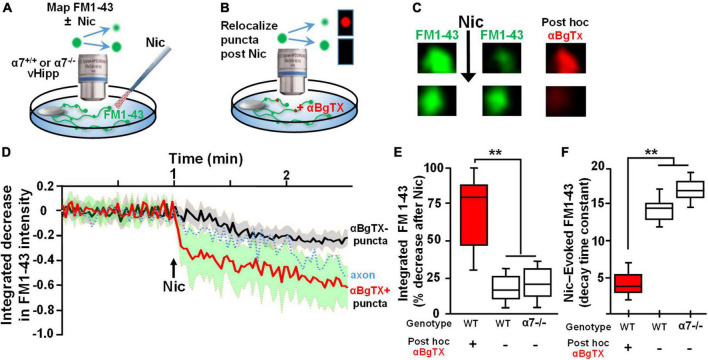
Re-localization of α7* nAChRs at individual puncta along ventral hippocampal axons following nicotine-induced FM1-43 de-staining. **(A,B)** Schematics of the experimental configurations (see Figure 1 legend for illustrative details). **(A)** vHipp from individual α7+/+ or α7–/– mice were plated as thinned micro-explants and were loaded with FM1-43, as described in Section “Materials and methods.” The dynamics of nicotine-induced release were assayed as FM1-43 de-staining along vHipp axons. **(B)** After recording the effects of nicotine (1 μM, 1 min)-induced FM1-43 de-staining, the vHipp axons were labeled for surface α7* nAChRs with αBgTx–Alexa 594. Individual FM1-43 “puncta,” including both those that had and those that had not previously shown nicotine-induced FM1-43 de-staining were re-localized after αBgTx–Alexa 594 labeling. **(C)** Representative micrographs of sample puncta, one where nicotine had induced robust de-staining (top) and another where nicotine had little effect on the FM1-43 signal (bottom). Subsequent re-localization of these puncta after αBgTx–Alexa 594 labeling, revealed co-localization of αBgTx at sites that had shown strong nicotine-induced FM1-43 de-staining (top), in contrast at sites with little or no αBgTx staining, nicotine had little or no effect on the FM1-43 signal (bottom). **(D)** Pooled traces of the time course of FM1-43 de-staining in response to nicotine for individual puncta, subsequently re-localized as αBgTx positive (red) vs. αBgTx negative (black). The 30 individual puncta that were re-localized after recording of FM1-43 destaining and that were found to be αBgTx Alexa 594 positive, are averaged before and after nicotine application (red line, SD: green shading; *n* = 30, 3; see Table 1 for details). The black line shows the averaged de-staining time course of 20 other FM1-143 sites that lacked *post hoc* αBgTx–Alexa 594 co-labeling (*n* = 20,3). The dashed trace in the middle is the time course of nicotine-induced FM1-43 de-staining including all the puncta along a vHipp axon (i.e., including both those with and without *post hoc* αBgTx–Alexa 594 co-labeling). **(E)** Box plots of pooled data of the decrease in FM1-43 staining in α7+/+ v Hipp axons (*n* = 60 FM1-43 puncta; three separate experiments; *n* = 60;3) with *post hoc* surface αBgTx–Alexa 594 labeling and in α7–/– vHipp axons (*n* = 46; 4). Puncta where nicotine elicited robust de-staining of FM1-43 corresponded to sites of positive *post hoc* staining for surface αBgTx–Alexa 594, consistent with local expression of α7* nAChRs (>80% decrease). In contrast, weak de-staining of FM1-43 at distinct sites, even along the same axons, was comparable to the partial de-staining seen along α7–/– vHipp axons and were not co-localized with *post hoc* labeling for surface αBgTx [<25% decrease, quantified from 60 *post hoc* αBgTx–Alexa 594 negative puncta. **p < 0.01. All data were first tested for distribution normality by the test(s) listed in Table 1 prior to statistical analysis]. **(F)** Box plot of pooled data for the decay time constants of nicotine-induced FM1-43 de-staining at subsequently re-localized sites along α7+/+ vs. α7–/– vHipp axons. The decay time constants at sites labeled by αBgTx, consistent with local surface α7* nAChRs (∼4 s, *n* = 60, 3) were significantly different from the decay time constants at sites along α7+/+ vHipp axons that lacked *post hoc* αBgTx staining (∼14 s; *n* = 60, 3; **p < 0.001). The αBgTx-negative puncta had destaining time constants comparable to the τ values at FM1-43 clusters along α7–/– vHipp axons (τ ∼ 16 s, *n* = 46, 4; see Table 1 for details and statistical tests).

To assess the spatial relationship between α7* nAChRs and sites of nicotine induced FM1-43 de-staining, we used αBgTx conjugated to Alexa 594 to visualize surface α7* nAChRs following nicotine-induced FM1-43 de-staining (Figures 2A,B). Over 80% of individual puncta along vHipp axons at which nicotine induced robust FM1-43 de-staining also stained with αBgTx-Alexa 594 indicating the presence of surface α7* nAChRs (Figure 2C). These double labeled vesicle clusters showed rapid and nearly complete (time constant ∼4 s, 80% decrease in FM1-43; red trace in Figure 2D) nicotine induced FM1-43 exocytosis. In contrast, vesicle clusters that were not stained with αBgTx –Alexa 594 showed significantly slower (time constant of ∼14.0) and only partial (<25% intensity decrease) de-staining (trace in black) (Figures 2D–F); values that were very similar to those recorded from α7 knock-out axons. The effect of nicotine on neurotransmitter vesicle release showed two distinct responses – an α7* nAChR dependent response that was associated with close apposition of surface α7* nAChRs to the sites of release and that was kinetically distinct from the second non-α7* nAChR, presumably α4β2* nAChR (Zhong et al., 2013), mediated response.

**FIGURE 3:**
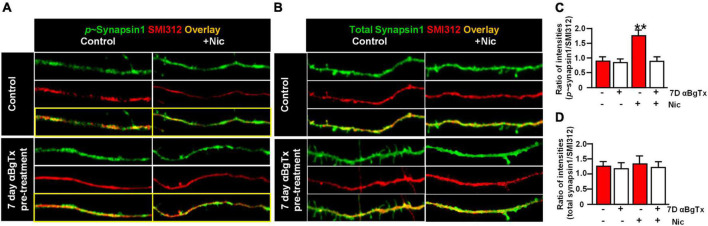
α7* nAChRs-mediate nicotine-induced phosphorylation of Synapsin1 along ventral hippocampal axons. Ventral hippocampi were obtained from individual α7+/+ pups and plated as thinned micro-slices to optimize axon outgrowth as described in detail in Figure 1 legend and in Section “Materials and methods.” A subset of these cultures was treated for 7-days with αBgTx (100 nM). Following acute exposure to nicotine (1 μM, 1 min), all samples were fixed, permeabilized, and labeled with antibodies recognizing phospho-Synapsin1 or total Synapsin1, and axonal neurofilaments using a “pan axonal” marker: SMI312. **(A)** Representative micrographs of vHipp axons (SMI312, red) from α7+/+ mice: no treatment control (top) vs. vhipp axons from α7+/+ mice following a 7-day, *in vitro* exposure to 100 nM αBgTx (bottom). These cultures were than subject to incubation under either control *or* + acute nicotine (1 min) treatment. After 5 min cultures were fixed, permeabilized and the distribution of phospho-synapsin1 was visualized (green) along vHipp axons; control (left) vs. 1 min after nicotine (right, scale bar: 10 μm). Note that the nicotine elicited increases in phosphorylated Synapsin1 seen along the α7+/+ control vHipp axons (top, right) was not detected in 7 D, chronic αBgTx treated conditions (right bottom). **(B)** Representative micrographs of vHipp axons (SMI312, red) from α7+/+ control (top) or α7+/+ following a 7-day exposure of αBgTx (bottom) prior to acute control vs. nicotine treatment. After fixation and permeabilization, the distribution of total Synapsin1 (green) was visualized along vHipp axons (SMI 312; red); Control (left) vs. 1 min treatment with acute nicotine (right, scale bar: 10 μm). There are no differences in total Synapsin1 or in SMI 312 labeling with (left, bottom) or without (left, top) 7-day pre-treatment with αBgTx. Nicotine did not increase total Synapsin1 along α7+/+ control vHipp axons (top, right) and the 7 D, chronic αBgTx treated vHipp axons (bottom, right). **(C)** Pooled and quantified data from experiments as shown in **(A)**. Twenty axon segments per condition were assayed in three separate experiments (four conditions; total *n* = 80; 3). Phospho-Synapsin1 immunofluorescent intensities along vHipp axons were quantified as a ratio of p-Synapsin1/SMI312 per 100 μm axon length. With no pre-treatment, the p-Synapsin1/SMI 312 ratio was 0.88 ± 0.12 before nicotine exposure and ∼2-fold higher after acute nicotine application (1.74 ± 0.18). In contrast, the 7-day pretreatment with αBgTx, a specific α7* nAChRs antagonist, blocked nicotine induced phosphorylation of Synapsin1 (0.85 ± 0.09 before vs. 0.90 ± 0.16 after nicotine) without altering the baseline ratios. All data were first tested for distribution normality by the test(s) listed in Table 1 prior to statistical analysis. Data represent the mean ± SEM, **p < 0.01, One-Way ANOVA *Post Hoc* Tests. See Table 1 for all n values and statistical tests. **(D)** Pooled and quantified data from experiments as shown in **(B)** (*n* = 80, 3). Total Synapsin1 immunofluorescent intensities along vHipp axons were quantified as ratio of total Synapsin1/SMI312 per 100 μm axon. The ratio of total Synapsin1 to SMI312 was unaffected by acute nicotine exposure whether under control conditions (1.28 ± 0.16 before vs. 1.39 ± 0.18 after nicotine application) or if vHipp axons were pre-treated with a 7-day exposure to 100 nM αBgTx (1.21 ± 0.18 before vs. 1.26 ± 0.18 after nicotine application). All data were first tested for distribution normality by the test(s) listed in Table 1 prior to statistical analysis. Data represent the mean ± SEM, *p* > 0.05, One-Way ANOVA *Post Hoc* Tests. See Table 1 and **(D)** panel specific data for all n values and statistical tests.

A dynamic synapsin 1 phosphorylation/dephosphorylation cycle is essential for maintaining synaptic vesicle organization and abundance in axon terminals (Bykhovskaia, 2011; Shupliakov et al., 2011; Patzke et al., 2019). To determine if synapsin 1 phosphorylation is a target for α7* nAChRs signaling, we examined the effect of acute nicotine or chronic αBgTx treatment on axonal levels of phospho-synapsin 1. vHipp micro-slices from α7+/+ or α7+/+ treated for 7-days with αBgTx, were exposed to nicotine (1 μM, 1 min), fixed, permeabilized, and stained with antibodies recognizing phospho-Synapsin 1 (serines 62 and 67; Figure 3A, green) or total synapsin 1 (Figure 3B, green), as well as pan-axonal (SMI312; red) antibodies. Levels of phospho-synapsin 1 increased following nicotine, in an α7* nAChR-dependent manner (blocked by αBgTx; Figures 3A,C; *p* < 0.01). Neither nicotine nor αBgTx affected the total level of axonal synapsin 1 (Figures 3B,D). These data are consistent with α7* nAChR mediated signaling *via* synapsin 1 phosphorylation, contributing to local modulation of synaptic vesicle cycling.

### α7* nicotinic acetylcholine receptors are required for sustained nicotine induced glutamate release

**FIGURE 4:**
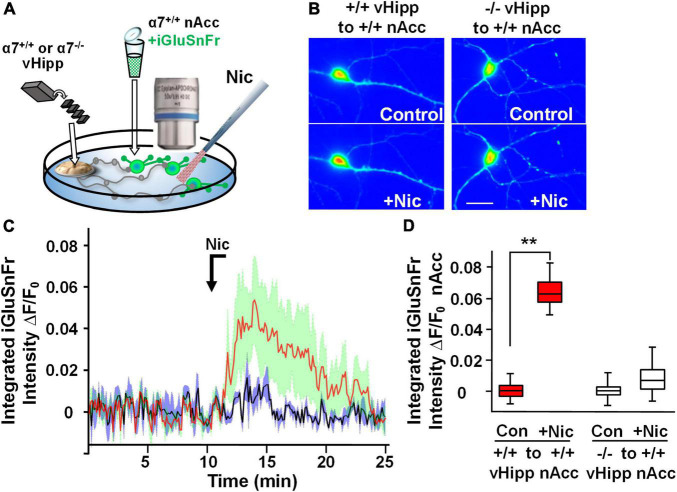
Hippocampal axonal α7* nAChRs are required for nicotine-evoked glutamate release at sites of ventral hippocampal – nucleus accumbens interaction. Nicotinic modulation of glutamatergic signaling was examined in gene chimeric co-cultures of ventral hippocampus (vHipp – plated as thinned microexplants) and nucleus accumbens neurons (nAcc – plated as dispersed neurons). **(A)** Schematic diagram of experimental configuration. Neurons, dispersed from P1, α7+/+ mouse nAcc were plated in media containing iGluSnFr (AAV_9_.hSyn. *iGluSnFr.WPRE*.SV40) for 24 h prior to washout and addition of vHipp microexplants. Individual WT (α7+/+) or α7 knock out (α7–/–) mice were used for each plating of vHipp micro-explants [prepared as previously described (Zhong et al., 2008)]. After plating of the microexplants, additional incubation in reduced media volume was allowed for explant thinning. After 7–10 days of co-culture, iGluSnFr fluorescence activity in the dispersed nAcc neurons was used as a read out of glutamate release. **(B)** Representative spinning disk confocal images of iGluSnFr fluorescence in nAccs neurons recorded from α7+/+ or α7–/– vHipp to α7+/+ nAcc co-cultures before (top) and after (bottom) nicotine (1 μM, 1 min) application. The relative signal intensity in the nAcc neurons, indicated by a pseudo color scale, is equivalent to significant increases in nicotine induced- glutamate release in co-cultures where vHipp is from α7+/+ mice (orange/red). In contrast, nicotine had little effect on glutamate release in co-cultures with α7–/– vHipp: Scale bar: 10 μm. **(C)** Averaged traces of nicotine induced changes in normalized somatic iGluSnFr intensity as a function of time. The red line shows the averaged somatic iGluSnFr intensity time course (±SD in green shading), from six examples of nAcc neurons from α7+/+ mice, cocultured with α7+/+ vHipp explants, before and after nicotine (1 μM, 1 min) application. The black line shows the averaged somatic iGluSnFr intensity time course ±SD in gray shading from five examples of nAcc neurons from α7+/+ mice co-cultured with α7–/– vHipp explants before and after nicotine application. Note that the nicotine induced glutamate release recorded in the +/+ vHipp to +/+ nAcc co-cultures was not seen in –/– vHipp to +/+ nAcc co-cultures. **(D)** Box plots of pooled data: Nicotine increased integrated iGluSnFr intensity (Δ*F/F_0_*) in co-cultured nAcc neurons with α7+/+ vHipp innervation (**p < 0.01, eight neurons/condition three separate experiments) but not in nAcc neurons co-cultured with α7–/– vHipp innervation (*p* > 0.05, 11 neurons/condition; 4 separate experiments). All data were first tested for distribution normality by the test(s) listed in Table 1 prior to statistical analysis. See Table 1 for all *n* values and statistical tests.

Modulation of the release of glutamate by activation of presynaptic nAChRs is a prevalent mechanism of nicotinic facilitation of glutamatergic transmission in the CNS. To visualize α7* nAChRs modulation of glutamate release, we utilized the genetically encoded fluorescent glutamate indicator, iGluSnFr. Dispersed medium spiny neurons (MSNs) from wild type nAcc were plated and transfected with iGluSnFr before the addition of vHipp micro-slices prepared from either wild type or α7 knock-out pups. We monitored changes in fluorescence of iGluSnFr expressed on the disperse nAcc MSNs following application of nicotine to the co-cultures (Figure 4A). A 1 min nicotine (1 μM) application resulted in sustained increase of iGluSnFr fluorescence intensities around nAcc MSNs soma co-cultured with WT (α7+/+) vHipp (Figures 4B–D; *p* < 0.01). No significant increase in iGluSnFr fluorescence intensity was seen in WT MSNs cultured with α7 knockout vHipp (Figures 4B–D). These data, consistent with our previous electrophysiological results (Zhong et al., 2008), demonstrate that presynaptic α7* nAChRs mediate the sustained responses to nicotine.

**FIGURE 5:**
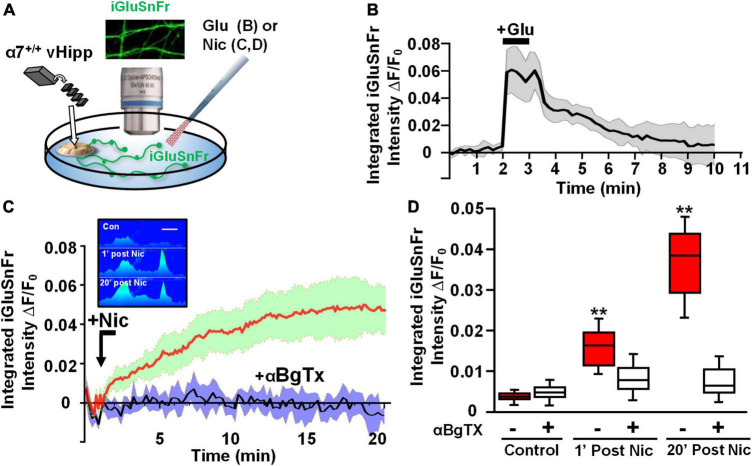
Axonal α7* nAChRs are required for nicotine-evoked glutamate release along ventral hippocampal projections. Nicotinic modulation of glutamatergic signaling along vHipp axons. **(A)** Schematic diagram of experimental configuration, see Section “Materials and methods.” In these studies, vHipp from individual WT (α7+/+) mice were plated as thinned micro explants in iGluSnFr (AAV9.hSyn.*iGluSnFr.WPRE*.SV40) containing culture media (see Section “Materials and methods”). After 7–10 days *in vitro*, iGluSnFr fluorescence signals along the vHipp axonal projections were recorded as an assay of glutamate release. Representative images of iGluSnFr expressing axons are shown. **(B)** iGluSnFr fluorescence intensities were calculated from spinning disk confocal images of α7+/+ vHipp axons, collected every 10 s for 10 min and quantified as a normalized integrated intensity at each time point. The percentage changes in normalized integrated intensity at individual 1-μm spots along vHipp axons were plotted vs. time. Traces from α7+/+ vHipp axons are shown before, during and after glutamate-application (averaged of two recordings from two separate experiments). Application of exogenous glutamate (100 μM, 1 min) increased iGluSnFr fluorescence intensity by ∼7%; this increase is maintained during glutamate application and decays to baseline over the next 5 min after glutamate wash out. Glutamate applications at lower concentrations did not show significant increase of iGluSnFr fluorescence intensities (data not shown). **(C)** Averaged traces of normalized iGluSnFr intensity along α7+/+ vHipp axons before and after exposure to nicotine. Under normal conditions a 1 min exposure to nicotine elicited a robust increase in iGluSnFr signal along the vHipp axon, consistent with detection of local release glutamate (red line; SD = green shading, *n* = 20, 3). The effects of αBgTx treatment – which blocks all detectable nicotine induced iGluSnFr signal – is shown by the averaged traces in black (with SD shaded blue; *n* = 20, 3). Inset in **(C)**, representative spinning disk confocal iGluSnFr images recorded along the same axon segment before (top), 1 min after (middle), and 20 min after (bottom) nicotine (1 μM, 1 min) application (pseudo color scale for release profile; inset scale bar 5 μm). **(D)** Box plots of pooled data of iGluSnFr fluorescence intensity before and after acute nicotine application. Pre-incubation with αBgTx (100 nM, 5 min) had no detectable effect on iGluSnFr baseline (Control *n* = 20,3 vs. +αBgTx: 20, 3; *p* > 0.05). Nicotine (1 μM, 1 min) elicited a significant increase in integrated iGluSnFr intensity with both an acute (1 min, **p < 0.01) and sustained (20 min; **p < 0.01) component, which were largely blocked by inclusion of the α7 receptor antagonist, αBgTx. All data were first tested for distribution normality by the test(s) listed in Table 1 prior to statistical analysis. See Table 1 for all *n* values and statistical tests.

Next, we took advantage of the ability of iGluSnFr to sum glutamate signals (Helassa et al., 2018) to image nicotine modulated release sites along vHipp axons. We infected vHipp micro-explants with AAV.hSyn.iGluSnFr and confirmed axonal iGluSnFr expression by imaging responses to glutamate (100 μM, 1 min) added to the cultures. iGluSnFr Δ*F*/*F*_o_ increased from ∼0 to 0.07 during the glutamate applications and returned to baseline following washout (Figures 5A,B). Having established that we could record changes in iGluSnFr fluorescence along vHipp projections, we used iGluSnFr to assay the effect of nicotine application on glutamate release. Nicotine application (1 μM, 1 min) increased iGluSnFr fluorescence intensities (Δ*F*/*F*_o_ ∼4%). After washout of nicotine, iGluSnFr fluorescent intensity continued to increase and then remained elevated for at least 20 min (baseline vs. 1 min post-nicotine: *p* < 0.01; baseline vs. 20 min post-nicotine, *p* < 0.01). This robust and sustained response was abolished by preincubation with alpha-bungarotoxin (αBgTx), an α7* nAChRs selective antagonist (Figures 5C,D). Although we are plotting the integrated fluorescence intensity along vHipp axons in Figure 5C, the increase in signal was focal (Figure 5C, inset), consistent with stimulation of glutamate release from discrete sites.

### Endogenous acetylcholine induces sustained glutamate release along vHipp axons

**FIGURE 6:**
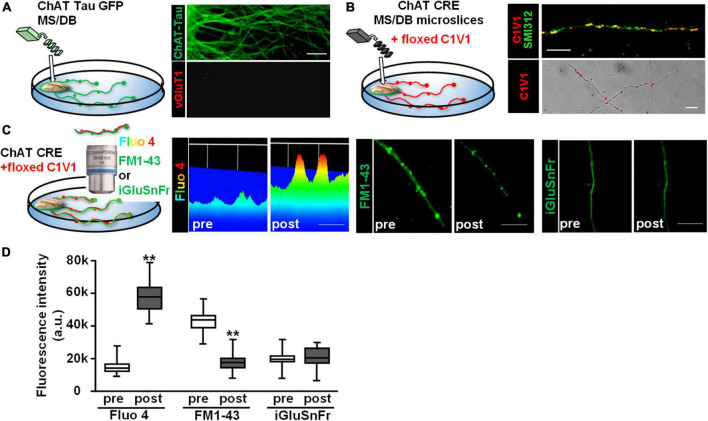
Optogenetic stimulation of C1V1 expressing MS/DB cholinergic projections elicits local Ca^2+^ influx and FM1-43 de-staining of cholinergic axons. **(A)** Schematic diagram of experimental configuration for assessing the outgrowth of cholinergic projections from thinned micro-explants of medial septum/diagonal band (MS/DB) from ChAT-tau-GFP mice. Representative confocal images of ChAT-tau-GFP fibers indicate that the projections from MS/DB micro-explants maintained *in vitro* are cholinergic, i.e., labeled with ChAT tau-GFP (in green, **A** right top) and are not marked by glutamatergic probes such as vGluT1 (**A** right bottom). **(B)** Schematic of MS/DB slices obtained from ChAT-IRES-Cre mice (ChAT CRE) plated as micro thinned explants and then labeled with the floxed red-shifted variant of the optogenetic channel rhodopsin, C1V1 (pAAV-Ef1a-DIO C1V1 (t/t)-TS-mCherry) by *in vitro* infection. After 7–10 days *in vitro*, C1V1 labeled MS/DB projections were also labeled with pan axonal marker (SMI312). A representative confocal image shows C1V1 labeled processes detected as red puncta (**B** right bottom, scale bar: 10 μm). These red puncta are detected along axonal (SMI312 positive; green) projections consistent with C1V1 labeling of cholinergic axons (**B** right top, scale bar: 10 μm). **(C)** (Left panel) Schematic diagram of experimental set up of MS/DB slices from ChAT-IRES-Cre mice, labeled *in vitro* with the floxed C1V1. (Middle and Right panels) After 7–10 days *in vitro*, optogenetic stimulation of C1V1 evoked cholinergic signaling along MS/DB axons as verified by Ca signaling (Fluo4) and FM1-43 destaining (**C** left and middle micrographs). Representative spinning disk confocal images before (pre) and after (post) C1V1 opto-stimulation (100× 5 ms flashes at 594 nm, 10 Hz × 10 s) showing examples of Ca^2+^ signaling (assayed with Fluo 4; pseudo color heat map of individual axons, scale bar: 5 μm) and vesicle release (assayed by FM1-43 de-staining, scale bar: 10 μm). Selective cholinergic activation (not glutamatergic) was confirmed in assays of glutamate release by iGluSnFr expression. (Far right panel) There was no evidence for release of glutamate from the same MS/DB cholinergic axons before or after C1V1 mediated optogenetic activation. Scale bar: 10 μm. **(D)** Boxplot of pooled data from 20 axon segments per condition in 4 separate experiments. Photo-stimulation of C1V1 expressing cholinergic projections elicited an ∼3× increase in axonal Ca^2+^ signaling (assayed as increase in Fluo 4 fluorescence intensity; *n* = 20; 4; **p < 0.001) and about a twofold increase in vesicle fusion and release (assayed from de-staining of FM1-43 fluorescence intensity; *n* = 20, 4; **p < 0.001). Under the same conditions there were no significant changes in iGluSnFr fluorescence intensity (*n* = 20, 4; *p* > 0.05). All data were first tested for distribution normality by the test(s) listed in Table 1 prior to statistical analysis. See Table 1 for all *n* values and statistical tests.

We next sought to determine whether endogenously released ACh would also stimulate glutamate release from vHipp axons. To test this, we co-cultured vHipp explants with micro-slices of the medial septum/diagonal band of broca (MS/DB). First, we stained MS/DB micro-slices from ChAT-tau-GFP mice cultured alone with vGluT1 antibodies to confirm that these MS/DB micro-slices extend cholinergic (GFP^+^) but not glutamatergic (vGluT1^–^) projections (Figure 6A). Next, we infected MS/DB micro-slices from ChAT-IRES-Cre mice with AAV-Ef1a-DIO.C1V1.(t/t)-TS-mCherry to express the red-shifted channel rhodopsin variant, C1V1, only in cholinergic neurons (Figure 6B, pan-axonal marker in green, C1V1 in red). We monitored the effects of focal optogenetic stimulation (594 nm LED light, 5 ms pulses at 10 Hz for 10 s) of cholinergic fibers in three ways: (1) axonal Ca^2+^ dynamics following loading of cultures with Fluo 4; (2) FM1-43 vesicle de-staining; and (3) we looked for evidence of glutamate release by co-expressing iGluSnFr (Figures 6C,D). Photo-stimulation of C1V1 expressed along MS/DB cholinergic axons increased axonal calcium levels (*p* < 0.001) and induced vesicular release of FM1-43 (*p* < 0.001). We did not detect any release of glutamate from these explants following C1V1 stimulation (iGluSnFr fluorescence intensity did not change, Figure 6D).

**FIGURE 7:**
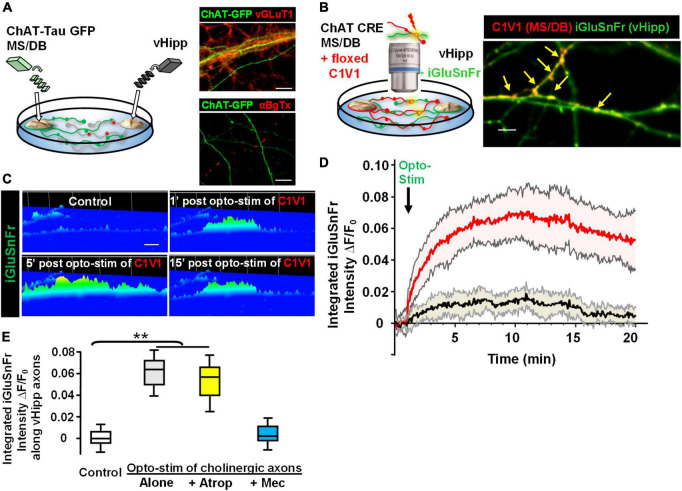
Opto-stimulation of C1V1-expressing MS/DB cholinergic projections elicits glutamate release along co-cultured vHipp axons *via* nAChRs. Endogenous cholinergic modulation of glutamatergic signaling was examined in co-cultures of septal (MS/DB)-ventral hippocampal micro explants, focusing on sites of axo-axonic interactions. **(A)** Left: Schematic diagram of experimental configuration: vHipp, thinned micro-explants were prepared from WT mice and plated as delineated in Section “Materials and methods.” Twenty-four hours post plating, MS/DB cholinergic explants were prepared from ChAT-tau-GFP mice. After 7–10 days *in vitro*, these septal-hippocampal co-cultures were labeled for vGluT1 (anti-vesicular glutamate transporter 1) and/or surface α7* nAChRs (αBgTX-Alexa 594). **(A)** (Right, Top) Representative confocal images of GFP labeled cholinergic projections from MS/DB explants of ChAT-tau-GFP mice (in green), intermingled with vGluT1^+^ (red) projections from vHipp. Scale bar: 10 μm. (Right, Bottom) Different representative images of GFP labeled cholinergic projections from MS/DB explants of ChAT-tau-GFP mice (in green), intermingled with αBgTX^+^ labeled (red) projections from vHipp. Note that αBgTX^+^ (α7* nAChRs) labeling was only detected along GFP negative, vHipp axons, but not along GFP positive MS/DB axons. Scale bar: 10 μm. **(B)** Left: Schematic diagram of experimental configuration used to assess endogenous ACh-evoked glutamate release at axo-axonic contacts between MS/DB and vHipp. Micro-explants of vHipp were prepared from WT mice and plated as described in Section “Materials and methods.” To detect glutamate release from glutamatergic axons, iGluSnFr (AAV9.hSyn. *iGluSnFr.WPRE*.SV40) was added to the culture media to infect vHipp neurons for 24 h. Following washout of iGluSnFr, the explants of MS/DB from ChAT-IRES-Cre mice were plated on the same coverslip (Zhong et al., 2015). Cholinergic neurons were then selectively labeled with the floxed red-shifted variant of channel rhodopsin C1V1 (pAAV-Ef1a-DIO C1V1 (t/t)-TS-mCherry) by subsequent infection *in vitro*. After 7–10 days *in vitro*, the co-cultures of iGluSnFr labeled, glutamatergic-vHipp axons with C1V1-labeled cholinergic axons were subjected to concurrent optogenetic stimulation and recording of glutamate release. **(B)** Right: Representative confocal image of chimeric co-culture of C1V1 (in red) expressing ChAT-Cre MS/DB projections with multiple sites of contact (indicated by yellow arrows) with iGluSnFr expressing WT vHipp axons (green). Scale bar, 10 μm. **(C)** Optogenetic stimulation of endogenous ACh release from MS/DB axons induced glutamate release from contacted vHipp axons. Representative spinning disk confocal iGluSnFr images recorded from WT vHipp axons before and after photo-stimulation (100 flashes of 594 nm light, 10 Hz × 10 s; 5 ms duration) of C1V1 expressed MS/DB axons (spatio-temporal distribution of iGluSnFr signal is represented in pseudo color. Scale bar: 5 μm). **(D)** Averaged traces of the time course of changes in glutamate release (normalized iGluSnFr intensity) along vHipp axons following optogenetic stimulation of endogenous ACh release from MS/DB axons. Averaged changes in iGluSnFr intensity as a function of time in 21 vHipp axons from 3 separate experiments before and after photo-stimulation (*n* = 21,3: red; SD shown in shaded pink). The vHipp axons were contacted by co-cultured MS/DB axons expressing C1V1 (as the yellow arrow shown in **B**). The black line shows the averaged changes in iGluSnFr intensity time course from 9 vHipp axons without cholinergic input before and after photo-stimulation (*n* = 9, 3 black line; SD gray shading). Although these vhipp axons were also co-cultured with C1V1 expressing MS/DB micro-explants, no points of C1V1 contact were evident. Note that the robust, opto-stimulated, glutamate release seen at cholinergic axon-vHipp axonic contacts was not detected along vHipp axons that were not contacted by C1V1 expressing cholinergic axons. **(E)** Box plots of pooled data showed photo-stimulation of C1V1 expressing MS/DB axons under control conditions (**p < 0.01, *n* = 30, 6 co-cultures) or with concurrent incubation with atropine (0.5 μM, *n* = 10,3 **p < 0.01) evoked a significant and sustained increase in the integrated iGluSnFr fluorescence intensity along contacted vHipp axons. In contrast, photo-stimulation in the presence of mecamylamine (10 μM, *n* = 10, 3, *p* > 0.05), was without effect, consistent with nicotinic ACh receptor mediated, ACh-evoked glutamate release. All data were first tested for distribution normality by the test(s) listed in Table 1 prior to statistical analysis. See Table 1 and Figure **(E)** panel-specific data for all *n* values and statistical tests.

Next, we plated co-cultures of MS/DB (from ChAT-tau-GFP pups) and vHipp (from C57Bl6 pups) micro-slices and stained for either vGluT1 or surface α7* nAChRs. Cholinergic projections (in green) from MS/DB micro-slices intermingled with glutamatergic projections (in red) from WT vHipp micro-slices (Figure 7A, top). vGluT1 and α7* nAChRs positive staining were detected along vHipp projections (Figure 7A, bottom). We did not detect any α7* nAChRs along MS/DB projections using this micro-slice preparation.

Having established that in this co-culture preparation cholinergic and glutamatergic neurons were physically intermingled we next plated and infected WT vHipp micro-slices with AAV_9_-hSyn.iGluSnFr and then 1 day later added MS/DB micro-slices from ChAT-Cre animals to the same coverslips and infected the co-culture with AAV-EF1a.DIO.C1V1-mCherry (Figure 7B). This allowed us to quantify glutamate release along the vHipp axons (expressing iGluSnFr) following optostimulation of C1V1 (expressed in cholinergic neurons). We stimulated C1V1 (594 nm, 5 ms, 10 Hz for 10 s) and measured iGluSnFr fluorescence. Optogenetic stimulation of ACh release resulted in a sustained increase in iGluSnFr fluorescence along vHipp axons with close contacts with C1V1 expressed cholinergic axons (∼10-fold increase in Δ*F*/*F*_o_ for at least 15 min following light stimulation; Figures 7C,D, red trace). There were no changes in iGluSnFr fluorescence detected along vHipp axons without close contacts with C1V1 expressed cholinergic axons in the same cultures (Figure 7D, black trace). The vHipp axon iGluSnFr response following optostimulation of cholinergic axons was blocked by the selective nicotinic receptor antagonist mecamylamine, but not by the muscarinic receptor antagonist atropine (Figure 7E). These results demonstrate that brief stimulation of ACh release (10 s) from septal-cholinergic neurons can facilitate sustained (15 min) vHipp glutamate release *via* presynaptic nAChRs.

### α7* nicotinic acetylcholine receptor signaling regulates synaptic vesicle clustering along vHipp axons

**FIGURE 8:**
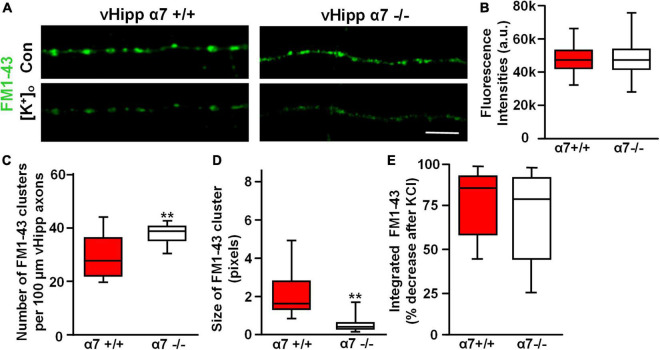
α7* nAChRs are required for synaptic vesicles clustering but not for depolarization induced FM1-43 de-staining along ventral hippocampal axons. **(A)** Micro-explants of vHipp from α7+/+ or α7–/– mice were plated for live imaging of axonal projections and assessment of FM1-43 de-staining as an assay of transmitter release. Representative micrographs of WT (α7+/+, **A**, left) and α7–/– (**A**, right) vHipp axons after loading with and washout of FM1-43 (green) under control conditions (**A**, top) and after depolarization with elevated extracellular K^+^ (**A**, bottom). Responses to depolarizing conditions are equivalent independent of genotype. Scale bar, 10 μm. **(B)** Box plot of pooled data from six separate vHipp explants cultures for each group showed equivalent initial FM1-43 staining after K^+^-dependent depolarization (assayed with total FM1-43 fluorescence intensities) between α7+/+ (*n* = 100; 5) and α7–/– (*n* = 92, 4) vHipp axons. **(C,D)** Comparison of the number and size of FM1-43 positive puncta along vHipp axons from α7+/+ (*n* = 100; 5) vs. α7–/– (*n* = 92, 4) mice. Box plot of pooled data comparing α7+/+ vs. α7–/– in terms of sites of release (FM1-43 puncta) reveals a significantly greater number of clusters (**C**, **p < 0.01) with an equally significant decrease in cluster size (**D**, **p < 0.01; cluster sizes were measured as continuous fluorescent pixels where 1 pixel – 0.267 μm) of FM1-43 positive puncta. All data were first tested for distribution normality by the test(s) listed in Table 1 prior to statistical analysis. See Table 1 and figure for all *n* values and statistical tests. **(E)** Box plots of pooled data on the effect of depolarization (elevated [K]_ext_) on FM1-43 destaining from both α7+/+ (*n* = 20,5) and α7–/– (*n* = 20,4) vHipp axons. The efficacy of K^+^-dependent depolarization on release along vHipp axons was assayed as the percent decrease of overall FM1-43 fluorescence intensity after increased [K]_ext_. The effect was equivalent regardless of genotype of the donor mice α7+/+ (*n* = 5) and α7–/– (*n* = 4). All data were first tested for distribution normality by the test(s) listed in Table 1 prior to statistical analysis.

Previously we reported that FM1-43 labeled puncta along α7−/− vHipp axons were more numerous but smaller than FM1-43 positive puncta along WT vHipp axons (Zhong et al., 2013). We found similar differences in our current study. Although FM1-43-labeling was equivalent in WT (+/+) (Figure 8A, left top) and α7 knockout (−/−) (Figure 8A, right top) vHipp axons, and showed no differences in the initial FM1-43 fluorescence intensities after K^+^-dependent loading (Figure 8B), the α7 knockout (−/−) vHipp axons had more (KO: 38.4 ± 4.2 clusters/100 μm vs. WT 27.8 ± 3.1 clusters/100 μm; *p* < 0.01) and smaller FM1-43 positive puncta (measured in fluorescence pixels) than the WT vHipp axons (KO: 0.4 ± 0.3 vs. WT: 1.78 ± 0.8; *p* < 0.01) (Figures 8C,D). Despite the differences in FM1-43 labeled puncta number, size and nicotine responsiveness, FM1-43 loading, and de-staining was equivalent in α7 knock out and WT axons. vHipp micro-slices from either WT or α7 knockout mice were loaded with FM1-43 and then KCl –induced de-staining of the readily releasable pool of vesicles was quantified. After high K^+^ (56 mM) application, the fluorescence intensity of all puncta along WT (+/+) vHipp axons (Figure 8A, left bottom) decreased by ∼85% (Figure 8E), reflecting depolarization-induced exocytosis of synaptic vesicles. The decrease in depolarization-induced fluorescence intensity of FM1-43 along α7 knockout (−/−) vHipp axons was the same as WT (Figure 8A, right bottom; Figure 8E).

**FIGURE 9:**
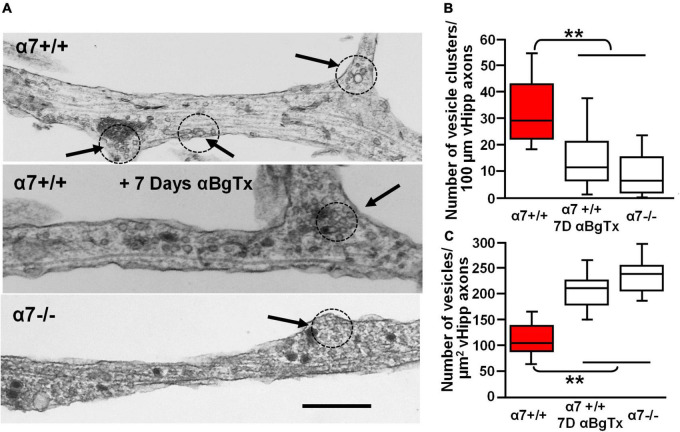
Electron microscopic examination of the role of α7* nAChRs in the organization and density of synaptic vesicles along ventral hippocampal axons. Ultrastructural analysis reveals defects in the number and organization of synaptic vesicle clusters along vHipp axons with either genetic deletion or pharmacological block of α7* nAChRs. **(A)** Representative electron micrographs of axons emanating from control α7+/+ vHipp micro-explants (Top), from α7+/+ vHipp micro-explants, treated for 7-days *in vitro* with the α7* nAChRs blocker, αBgTx (Middle), or α7–/– mice (Bottom). The arrows and dotted circles indicate the location of vesicle clusters (defined as ≥15 vesicles within less than a vesicle diameter of one another; scale bar, 500 nm). **(B)** Box plot of pooled data on the number of vesicle clusters/100 μm of axon from control α7+/+ vHipp (*n* = 20, 2) compared with α7+/+ vHipp post 7-day exposure of αBgTx (*n* = 20, 2) and with α7–/– (*n* = 20, 2). There was a significant decrease in the number of vesicle clusters along vHipp axons from either pharmacological (αBgTx) or genetic ablation (α7–/–) of α7* nAChRs compared with α7+/+ control; **p < 0.01. **(C)** Box plot of pooled data shows a significantly greater total number of vesicles per square micrometer vHipp axons from either pharmacological (α7+/+; post 7-day exposure of αBgTx) or genetic ablation (α7–/–) of α7* nAChRs compared with α7+/+ control; **p < 0.01.

To examine further the effect of α7* nAChRs on vesicle organization we used transmission electron microscopic imaging of ultrathin sections of vHipp micro-slice cultures with extensive axonal arbors. Varicosities containing clusters of synaptic vesicles (≥15 vesicles of 30–60 nm diameter, within less than a vesicle diameter of one another) were clearly seen along vHipp axons (Figure 9A). There were fewer clusters per 100 μm of α7 knockout (−/−) vHipp axon compared with WT (+/+) control (Figure 9B): 8 ± 5 per 100 μm of α7 knockout vHipp axon compared with 29 ± 8 per 100 μm of WT vHipp axon (*p* < 0.01). When we quantified the total number of vesicles, we found more per μm^2^ of α7 knockout (−/−) vHipp axon compared with WT (+/+) control (Figure 9C): 242 ± 8 per μm^2^ of α7 knockout vHipp axon compared with 108 ± 5 per μm^2^ of WT vHipp axon (*p* < 0.01). To confirm that these differences were directly related to loss of α7* nAChRs signaling, we prepared WT vHipp micro-slices and maintained them in the presence of αBgTx for 7 days prior to processing for electron microscopy. αBgTx blockade of α7* nAChRs decreased the number of vesicle clusters (Figure 9A, middle, Figure 9B) (12 ± 8 per 100 μm of vHipp axon, *p* < 0.01) and increased the total number of vesicles (Figure 9C) (218 ± 8 per μm^2^ of vHipp axon, *p* < 0.01) compared to WT vHipp axon. Taken together, these findings demonstrate that α7* nAChRs signaling regulates synaptic vesicle abundance and the organization of synaptic vesicles into distinct clusters.

## Discussion

In this report we demonstrate that α7* nAChRs localize to glutamatergic release sites along vHipp axons where they mediate glutamate release in response to either applied nicotine or ACh released from MS/DB cholinergic axons. We also demonstrate that the absence of functional α7* nAChRs (genetically deleted or chronically inhibited with αBgTx) alters the organization of synaptic vesicles along vHipp axons. These results provide evidence for a dual role of axonal α7* nAChRs during both the maturation of presynaptic-like specializations and in modulating glutamate release in mature projections.

The presence and potential functional role of pre-synaptic nAChRs in the regulation of glutamate release has been demonstrated with a variety of approaches from synaptosome preparations to *in vitro*, *ex vivo* and *in vivo* opto-physiology (Vidal and Changeux, 1989; McGehee et al., 1995; Gray et al., 1996; Threlfell et al., 2012; Kosillo et al., 2016). In the BLA, glutamatergic transmission is enhanced by nicotine or acetylcholine released following optogenetic stimulation of cholinergic terminals and is thought to underlie sustained changes in synaptic potentiation associated with fear learning (Jiang and Role, 2008; Jiang et al., 2013, 2016). In prior *in vitro* and *ex vivo* studies we have defined two distinct responses, transient increases in glutamate release mediated by non-α7* nAChRs and sustained changes in release that require α7*nAChRs. A limitation of these prior studies was our inability to visualize axonal nAChRs relative to sites of glutamate release. In this study we leveraged the power of micro-explant co-cultures to directly image aspects of nAChRs-mediated axonal modulation of glutamatergic glutamate release, both by using FM1-43 destaining and using an axonally expressed glutamate sensor, iGluSnFr (Marvin et al., 2013, 2018; Helassa et al., 2018).

### Contribution of non-α7* nicotinic acetylcholine receptors to nicotine induced glutamate release

α7* nAChRs localized to synaptic vesicle clusters mediate nicotine induced glutamate release. Both α4β2* and α7* nAChRs are associated with axo-axonic regulation of the release of many transmitters with studies of glutamate, GABA and dopamine being the most common (Dickinson et al., 2008; Livingstone et al., 2009; Threlfell et al., 2012). Non-α7*nAChRs (α4β2*nAChRs) mediate rapid axonal responses to nicotine (Zhong et al., 2008, 2013) in synaptic co-culture preparations as well as in response to ACh release in acute striatal slice (Kramer et al., 2022; Liu et al., 2022). Here we show that although nicotine treatment leads to general FM1-43 de-staining along vHipp axons, closer examination reveals two populations of FM1-43 labeled clusters that are distinguished both by the rate and degree of destaining, and by the proximity of surface α7* nAChRs. Clusters that destained rapidly and completely were associated with α7* nAChRs, whereas those that showed slower and incomplete de-staining were not (Figure 2). It is important to note that these latter release sites were not inherently dysfunctional – all FM1-43 sites rapidly and completely de-stained in response to K^+^ depolarization. In some instances, we found the two classes intermingled along the same axon segments (the two examples shown in Figure 2C were within ∼5 microns of each other). The α7* nAChR signals that lead to sustained responses are apparently spatially constrained and do not significantly spread within the axon. It is not clear what factors spatially restrain the α7* nAChR signals, but these findings underscore the importance of appropriate precise targeting of axonal α7* nAChRs.

FM1-43 marks sites of synaptic vesicle cycling but does not distinguish between different types of synaptic vesicles. We used the genetically encoded glutamate reporter, iGluSnFr, to probe α7*nAChR contribution more directly to glutamate release *per se* along vHipp axons. A brief exposure to nicotine increased iGluSnFr fluorescence along vHipp axons for tens of minutes; a response that required functional α7* nAChRs in the vHipp neurons (Figure 5). In contrast, glutamate application (100 μM, 1 min) increased iGluSnFr transiently with fluorescence returning to baseline levels with a *t*_1/2_ of 2–3 min. Given the reversibility of the iGluSnFr signal following glutamate wash-out, the continued rise in fluorescence after nicotine washout likely reflects continuing, elevated release of glutamate from the vHipp projections involving downstream intracellular signaling cascades.

The minimal non-α7*nAChR mediated response to nicotine, either in terms of FM1-43 destaining or iGluSnFr fluorescence, is difficult to interpret. Previously, using patch clamp recordings in vHipp – nAcc co-cultures, we demonstrated that nicotine increased mEPSP frequency *via* both a sustained α7* nAChR-dependent and a transient, α7 nAChR-independent mechanism (Zhong et al., 2008). We have also seen clusters of α4* nAChR on vHipp axons that were similar in frequency (cluster/100 μm axon) to α7* nAChR clusters (Zhong et al., 2013). One possibility is that transient increases in synaptic vesicle fusion and glutamate release, as measured by mEPSP frequency (Zhong et al., 2008) are insufficient to give a detectable FM1-43 or iGluSnFr signal under our imaging conditions. Alternatively, the full maturation of a non-α7* nAChR responsive pre-synaptic site might require interactions with post-synaptic targets. This would be an intriguing distinction but requires further study. In sum, our results are consistent with prior studies showing that transient nicotine activation of pre-synaptically localized α7* nAChRs results in sustained glutamatergic synaptic transmission (Jiang and Role, 2008; Zhong et al., 2008, 2013; Jiang et al., 2013, 2016).

#### Release of acetylcholine from medial septum/diagonal band axons elicits sustained glutamate release

Much of the literature exploring cholinergic modulation of synaptic transmission, especially with a focus on presynaptic mechanisms, has utilized pharmacological approaches – either nicotinic or muscarinic specific drugs, or applied ACh. We took advantage of this co-culture system to demonstrate that ACh released from cholinergic axons could directly activate presynaptic/axonal nAChRs leading to sustained facilitation of glutamate release (Figure 7). These responses were only seen in vHipp axons in close proximity to C1V1 expressing cholinergic axons – vHipp axons from the same explants but not in apposition to C1V1^+^ axons did not visibly respond to ACh release (red vs. black traces in Figure 7D). This is consistent with the formation of specialized axo-axonic sites of cholinergic signaling; utilization of this chimeric co-culture preparation opens the door for more in depth structural and functional studies of these axo-axonic interactions.

Stimulation of ACh release from cholinergic terminals within the basal lateral amygdala *ex vivo* (i.e., in acute slice preparations) results in a sustained increase probability of evoked glutamate release at cortical-amygdala synapses (Jiang et al., 2016). Optogenetic stimulation of ACh release in the BLA also results in increased BLA principal neuron firing, both in slice and *in vivo*, and is critical for the formation of associative memories (Jiang et al., 2016). Whether and how ACh modulation of pre-synaptic inputs affects firing is not clear; it is clear that ACh is able to modulate glutamate transmission in a behaviorally relevant manner (Jiang et al., 2016).

Dysfunction of the basal forebrain cholinergic system is an early feature of Alzheimer’s disease and is likely to contribute to impairments in memory formation and recall. To date there is little information about the extent to which presynaptic α7* nAChR signaling occurs in the human brain but both α7* and non-α7* nAChRs have been identified as potential targets for pathological Aβ oligomers (Wang et al., 2000; Liu et al., 2012; Lombardo et al., 2016). Aβ acts as both an agonist and antagonist of α7* nAChRs, depending on Aβ concentration (Liu et al., 2001; Chin et al., 2007; Cecon et al., 2019). If sustained axonal signaling is a critical feature of glutamatergic transmission *in vivo*, ACh-independent disruption in the timing and/or location of α7* nAChR-dependent axonal signaling as a result of changing Aβ levels, might contribute to cognitive impairment.

### Axonal α7* nicotinic acetylcholine receptor signaling contributes to glutamatergic axon maturation

Somato-dendritic α7* nAChRs have been implicated in development of glutamatergic synapses and of adult born glutamatergic neurons (Campbell et al., 2010; Lozada et al., 2012; Fernandes et al., 2014). We demonstrate in this report that axonal α7*nAChR function contributes to both the establishment of glutamatergic release sites and in modulating glutamate release. Taken together, these findings indicate that these receptors act in multiple compartments to regulate the development of some glutamatergic circuits. The contribution of α7* nAChR to neuron development raises several questions. First, what aspects of α7* nAChR functions are responsible for the effects on neuronal development? α7* nAChRs have the interesting feature of operating on two distinct levels: they are ligand-gated cation channels that contribute to depolarization on rapid time scales (milliseconds) and they activate metabotropic-like signaling by direct coupling to heterotrimeric G proteins (Nordman and Kabbani, 2014; Nordman et al., 2014; Kabbani and Nichols, 2018; King et al., 2018). Both mechanisms result in increased intracellular Ca^2+^ levels, and it is likely that calcium signaling is the major driver of α7* nAChR contributions to development. A second outstanding question is the identity of the developmentally relevant α7* nAChR agonist. In this study we demonstrate that adding the α7* nAChR antagonist, αBgTx, to the culture media mimics the effect of genetic deletion of α7. This points to some degree of on-going signaling in the absence of ACh. Whether this signaling is driven by choline, Lynx prototoxins (Miwa et al., 2019) or other agents remains to be determined.

The localized effects of axonal α7* nAChR on glutamate release underscore the importance of accurate targeting of these receptors to specific compartments. It is noteworthy that axonal/pre-synaptic targeting of α7*nAChRs is regulated by at least two signaling molecules – Nrg1 and Wnt – that play important roles in neurodevelopment (Farías et al., 2007; Hancock et al., 2008; Zhong et al., 2017). Variants in both CHRNA7 and NRG1 genes are associated with schizophrenia and related endophenotypes (Leonard et al., 2000; Munafò et al., 2006; Chen et al., 2009) and α7*nAChR levels are associated with NRG1 risk variants. The dual role of α7*nAChR in both glutamatergic axonal maturation and modulation of glutamatergic circuits, raises the possibility that the α7*nAChRs contribution to schizophrenia associated endophenotypes might reflect alteration in both functions.

## Data availability statement

The original contributions presented in this study are included in the article/supplementary material, further inquiries can be directed to the corresponding author.

## Ethics statement

This animal study was reviewed and approved by Stony Brook University Institutional Animal Care and Use Committee (#1618) NINDS ACUC (ASP#1490).

## Author contributions

CZ, DT, and LR conceived of the study, designed experiments, and prepared the initial draft of the manuscript. CZ and WA performed the experiments. CZ analyzed the data and prepared figures. All authors revised the manuscript, figures, and approved the submitted version.
